# The Unrecorded Injury from the Continued Use of Large Doses of Iron

**Published:** 1887-02

**Authors:** 


### *THE UNRECORDED INJURY FROM THE CONTINUED USE OF LARGE DOSES OF IRON.*

There can be no doubt about the usefulness of large doses of iron in many different stages of disorders, and cases are on record in which one to three drachms, or even sometimes as much as one and a half to two ounces, of the tincture of iron have been given daily in erysipelas; so also half ounce of tincture of iron has been given daily to young children, followed by satisfactory results.

In anæmia, which is often dependent upon dyspepsia, the best and quickest way of curing both dyspepsia and anæmia is the administration of large doses of the chloride and sulphate of iron. Large doses of iron act as an immediate stimulant, rapidly removing anæmia, and removing gastro-intestinal catarrh, and renew the appetite and digestion; but if continued for more than a week or two, they cause a gastro-intestinal catarrh of their own, and if this be not attended with diarrhœa, or if a purgative be not given, serious symptoms may ensue.

Dr. J. Strahan has had such an experience, and calls attention, in the *British Medical Journal* of September 18, 1886, to the danger of obstruction of the bowels which is apt to follow large doses of iron given for any length of time consecutively. The preparations which he has found to cause symptoms of obstruction are as follows, namely:

Ringer's pill of five grains of dried sulphate of iron, which equals nine grains of the ordinary sulphate, half-drachm and one-drachm doses of the tincture of mistura ferri composita P. B., made so strong as to contain eight or ten grains of sulphate of iron and carbonate of potassium in each dose. Any of these, if given three times, or even twice, daily, for from one to two weeks, and if natural or artificial diarrhœa do not in the meantime wash out the quantities of insoluble sulphide, will cause severe colic and constipation, which morphine and turpentine stupes seem unable even to alleviate, and which, it seems, would continue indefinitely, or till death, if not removed by a smart saline purge, or other means. Dr. Strahan has seen twenty-four hours of rhythmic agonizing pain, very like labor, with nearly constant vomiting, no sleep or ease after a grain of morphine by the mouth, the temperature rising to 101° Fahr., and great depression of all the powers of life, from a couple of weeks' course of Ringer's pill, which had done immense good to the system at large. This has not occurred once only, but dozens of times. As soon as ever a large dose of any saline—Dr. Strahan prefers an ounce of sulphate of magnesia—has well acted, all symptoms rapidly disappear. At this time, and for twenty-four hours after, the stools seemed composed half of dirty water and half of black sand. The author thinks this latter fact explains the pathology of the matter. In fact, it is a real obstruction—an acute one, too—by quantities of insoluble sulphide of iron. The quantity of this black sand, which comes away when severe constipation and colic have occurred, is surprising. It seems to be much more in quantity than the whole iron ingested, although the stools have been jet-black all along, through excreting the sulphide. So, it seems, it must lodge somewhere; perhaps in the cæcum and appendix. Then, when the iron begins to sicken the patient (gastric catarrh), the sulphide concretes by mucus, and forms an obstruction which brings matters to a crisis. The length of time that a patient can take large doses of iron depends on the state of the bowels; if they be kept or remain slightly loose, he does not suffer at all; if he becomes constipated, the iron soon sickens, and the horrible pains begin; if he were to take a smart saline purge once a week, he could go on indefinitely. The saline gives the quickest relief, because it produces such a vast exosmosis of water from the intestinal wall at all points as to dissolve the mucus which binds the sand, and then washes it out. In extreme constipation, it dissolves fæcal lumps in the same way, except when the masses are enormous. It is thus the best purgative where there is a stricture in the bowels, with dilatation above, as it melts the fæces into a fluid, when they can run through the narrow part.

It is also well known to be the only safe purgative if any inflammatory lesion exist, enteritis or peritonitis. Mr. Lawson Tait takes advantage of this property of salines in his modern treatment of peritonitis ensuing on abdominal section. The principle here seems to be that of giving true and perfect physiological rest by dissolving and washing out all irritants.

Another great advantage the salines have is that they cause no pain, no griping. A teaspoonful of Epsom salts every two or three hours will put an end to many a case of fæcal obstruction in old people, and that without pain, where croton oil and drachm-doses of jalap have only given rise to unendurable pain and collapse.

It cannot, of course, be said that large doses of iron will affect every one in the way described, but in the nature of things it seems to be highly probable. There is excellent reason for the practice of giving large doses of iron. Laache, in "The Relation of Recent Researches on Blood-Corpuscles to Anæmia and Leukæmia," (*Deutsche Med. Woch.*, October 23, 1884,) shows that in anæmia it may be both corpuscles and hæmoglobulin in proportionate quantity which are deficient, or, as in true chlorosis, while the red cells are diminished, the hæmoglobin is reduced out of all proportion to the loss of red cells. In fact, in the latter condition, what red cells remain are individually "chlorotic," are not red, or, at least, not red enough. Again, after bleeding, the red cells increase much more rapidly than the hæmoglobin, until, assuming it to be evenly distributed among the corpuscles, it has sunk to seventy per cent. of the healthy standard. Now, in such cases, the value of large doses of iron is seen. According to Laache, small doses of iron chiefly stimulate the numerical increase of red cells; large doses restore both the number of cells and the amount of hæmoglobin in each. This explains the difference which most men have noticed in practice between the large and the small dose.—*Therapeutic Gazette.*

